# From MRI to FEM: an automated pipeline for biomechanical simulations of vertebrae and intervertebral discs

**DOI:** 10.3389/fbioe.2024.1485115

**Published:** 2025-01-03

**Authors:** Kati Nispel, Tanja Lerchl, Gabriel Gruber, Hendrik Moeller, Robert Graf, Veit Senner, Jan S. Kirschke

**Affiliations:** ^1^ Institute for Neuroradiology, TUM University Hospital, School of Medicine and Health, Technical University of Munich, Munich, Germany; ^2^ Associate Professorship of Sport Equipment and Sport Materials, School of Engineering and Design, Technical University of Munich, Garching, Germany; ^3^ Bonescreen GmbH, Munich, Germany

**Keywords:** patient-specific, MRI, FEM, automated, spine, vertebra, intervertebral disc, meshing

## Abstract

**Introduction:**

Biomechanical simulations can enhance our understanding of spinal disorders. Applied to large cohorts, they can reveal complex mechanisms beyond conventional imaging. Therefore, automating the patient-specific modeling process is essential.

**Methods:**

We developed an automated and robust pipeline that generates and simulates biofidelic vertebrae and intervertebral disc finite element method (FEM) models based on automated magnetic resonance imaging (MRI) segmentations. In a first step, anatomically-constrained smoothing approaches were implemented to ensure seamless contact surfaces between vertebrae and discs with shared nodes. Subsequently, surface meshes were filled isotropically with tetrahedral elements. Lastly, simulations were executed. The performance of our pipeline was evaluated using a set of 30 patients from an in-house dataset that comprised an overall of 637 vertebrae and 600 intervertebral discs. We rated mesh quality metrics and processing times.

**Results:**

With an average number of 21 vertebrae and 20 IVDs per subject, the average processing time was 4.4 min for a vertebra and 31 s for an IVD. The average percentage of poor quality elements stayed below 2% in all generated FEM models, measured by their aspect ratio. Ten vertebra and seven IVD FE simulations failed to converge.

**Discussion:**

The main goal of our work was to automate the modeling and FEM simulation of both patient-specific vertebrae and intervertebral discs with shared-node surfaces directly from MRI segmentations. The biofidelity, robustness and time-efficacy of our pipeline marks an important step towards investigating large patient cohorts for statistically relevant, biomechanical insight.

## 1 Introduction

Diagnosing spinal disorders and their underlying causes has long posed a substantial challenge, as recently evidenced again by a study that revealed significant discrepancies in diagnoses from different MRI centers based on one single MRI (Herzog et al., 2017). To offer individual prevention strategies, estimate personal risks or plan patient-specific therapies, more information needs to be derived from medical images. One important step in this process is the analysis of spinal biomechanics, which can, for instance, be done by experimental measurements or numerical simulations such as multi-body simulation (MBS) and the finite element method (FEM). While experimental setups are often limited to few subjects, numerical simulations can more easily be realized patient-specifically, as well as in large datasets. The latter aspect bears the potential to deliver new pathophysiologic insights (Lerchl et al., 2024), for instance, relating certain spine characteristics to IVD degeneration. This could eventually result in better prediction of disease progression. However, analyzing large datasets requires two key components: patient-specificity and automation.

During the last decade, valuable achievements have been made towards this goal. Automated medical image segmentations have profited from the rise of machine learning (Sekuboyina et al., 2021; Moeller et al., 2024) and started to serve as a basic prerequisite for numerical models. Considering MBS, Lerchl et al. published an automated pipeline for the calculation of compression and shear forces between vertebrae in musculoskeletal models, including individualized body weight, spinal alignment, and the attachment points of ligaments and muscles (Lerchl et al., 2022). Geometries were derived from computed tomography (CT) images using machine learning (Lerchl et al., 2022; Sekuboyina et al., 2021). In contrast to the surface meshes defining rigid bodies in the MBS, FEM meshes require solid 3D meshes to calculate the inner stress and strain in vertebrae or intervertebral discs (IVD). The methods to derive patient-specific FEM meshes from medical imaging data can broadly be divided into voxel-based mesh generation, mesh morphing or surface mesh filling. Multiple approaches have been presented in the past decade, which apply one of these methods in conjunction with different grades of automation (Castro-Mateos et al., 2015; Castro-Mateos et al., 2016; Malandrino et al., 2015; Clouthier et al., 2023; Campbell and Petrella, 2015; Campbell et al., 2016; Caprara et al., 2021; Lavecchia et al., 2018).

In voxel-based models, segmented voxels directly serve as hexahedral elements of an FEM mesh (Castro-Mateos et al., 2016).

Mesh morphing is based on the deformation of an FE template mesh towards landmarks (Campbell and Petrella, 2015; Lavecchia et al., 2018; Caprara et al., 2021; Fasser et al., 2022) or complete surface meshes (Castro-Mateos et al., 2015; Caprara et al., 2021; Fasser et al., 2022) that were identified in individual medical images. The morphing process inherently involves striking a balance between accuracy and mesh quality. Non-conforming anatomies of IVDs and vertebrae can only be adapted by template meshes through large deformations. However, large deformations result in poor mesh quality, likely leading to convergence issues during the final FEM simulation (Malandrino et al., 2015). To minimize the displacements between a template and target mesh, recent approaches used statistical shape modeling (SSM) for their generation (Clouthier et al., 2023; Campbell and Petrella, 2015; Caprara et al., 2021; Castro-Mateos et al., 2015). However, ensuring sufficient mesh quality remains one of the main challenges in morphed patient-specific spine models.

These limitations do not apply to the filling approach, in which surface meshes are derived directly from segmentations in an initial step, which is often done by applying the marching cubes algorithm (Lorensen and Cline, 1987). Acquiring MRI scans involves capturing multiple slices of one to 3 mm thickness from various perspectives, partly leading to limited spatial resolution and voxels with heterogeneous properties. This inherent characteristic is translated directly to the generated surface meshes, limiting their accuracy and demanding subsequent smoothing (Molinari and Falcinelli, 2021). In the literature, limited information is provided on applied smoothing protocols. However, low resolution or artifacts like stair steps have been compensated using overall vertebra smoothing (Mönch et al., 2010; Mönch et al., 2013). This results in a loss of detail, whereby features like vertebra edges or osteophytes are smoothed to the point of disappearance, which we here refer to as oversmoothing. Consequently, calculated FEM stresses might underestimate actual stresses, potentially leading to the overlooking of wedge fracture predictions (Nispel et al., 2024). To avoid over-smoothing and enable accurate biomechanical results, selective smoothing algorithms have been introduced in recent studies (Nispel et al., 2024). Once smoothed, surface meshes are filled with volume elements, which is usually done manually and involves computer-aided design (CAD) software (El Bojairami et al., 2020; Lavecchia et al., 2018; Zadpoor and Weinans, 2015). Although for the morphing approach, efforts have been made towards automated, patient-specific model generation, no algorithm has yet been published to automate the filling approach.

With MRI images being more challenging to segment automatically, *inter alia* due to their subtle contrast variations, automatically generated spine FE models are mainly based on CT. They thus often include an approximation of the IVD shapes, neglecting anatomical characteristics such as bulge (Campbell and Petrella, 2015; Lavecchia et al., 2018; Caprara et al., 2021). In addition, material parameters for the IVD, which vary significantly depending on the degree of degeneration, cannot be extracted from CT images. Recent achievements in fully automated MRI segmentations opened the door to geometrically accurate patient-specific FEM models for vertebrae and IVDs including varying material definitions for soft tissue (Moeller et al., 2024; Graf et al., 2023).

However, the derivation of both IVDs and vertebrae poses another challenge: Contact modeling becomes more complex if the contact surfaces of both bodies are not equivalent or do not share nodes. This diminishes computational efficiency, potentially resulting in less accurate outcomes (Papadopoulos and Taylor, 1992) and hinders coupled MBS and FEM simulations (Nispel et al., 2023). To better understand the challenges of realizing shared contact surfaces, it is important to note that smoothing typically involves specific node translations for each body and can only be applied to bodies separately. Thus, smoothing steps need to be implemented in a specific order and with respect to the preservation of the adjacent body’s node positions (El Bojairami et al., 2020).

In summary, methods either automate the process using the morphing approach or rely on the filling approach in combination with a manual procedure. To the best of our knowledge, there is currently no existing approach that automates the filling approach.

Addressing the need for automated approaches to realize large dataset investigations, we here present the first pipeline to create and simulate patient-specific FEM models of vertebrae and IVDs with shared-node contact surfaces from MRI segmentations in a completely automated manner. Note that the focus of this work is on methodological development, particularly in automating model creation. As such, complex material models or biologically related parameters such as varying loading conditions or patient-specific weights are deliberately excluded from this study. The FEM simulation presented here serves solely to verify that the pipeline produces models capable of converging under simplified conditions, laying the groundwork for future biological model validation.

## 2 Methods

**FIGURE 1:**
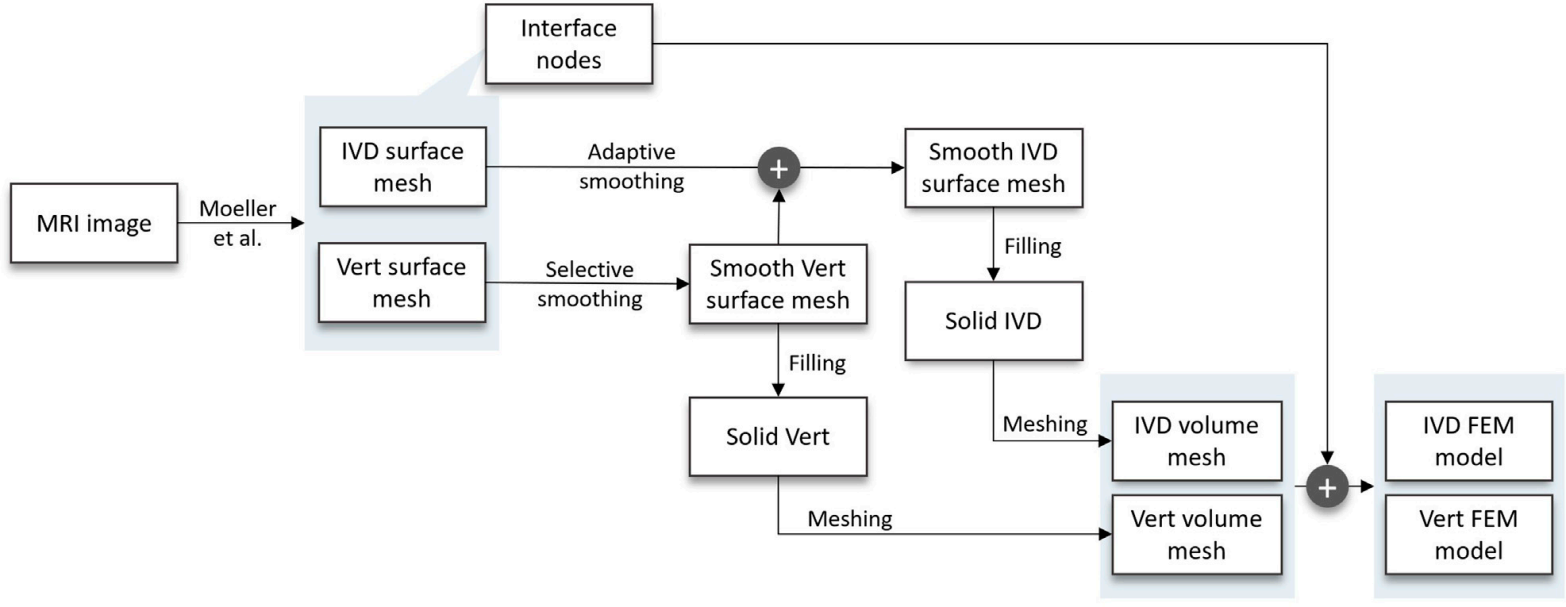
Flow chart of the developed pipeline, starting at the MRI scan and resulting in FEM models of the patients’ IVDs and vertebrae. In between, the following substeps are carried out: two distinct smoothing algorithms, surface mesh filling, volume meshing, and the inclusion of interface nodes in implementing an FEM model of the vertebra and IVD, respectively.

**FIGURE 2:**
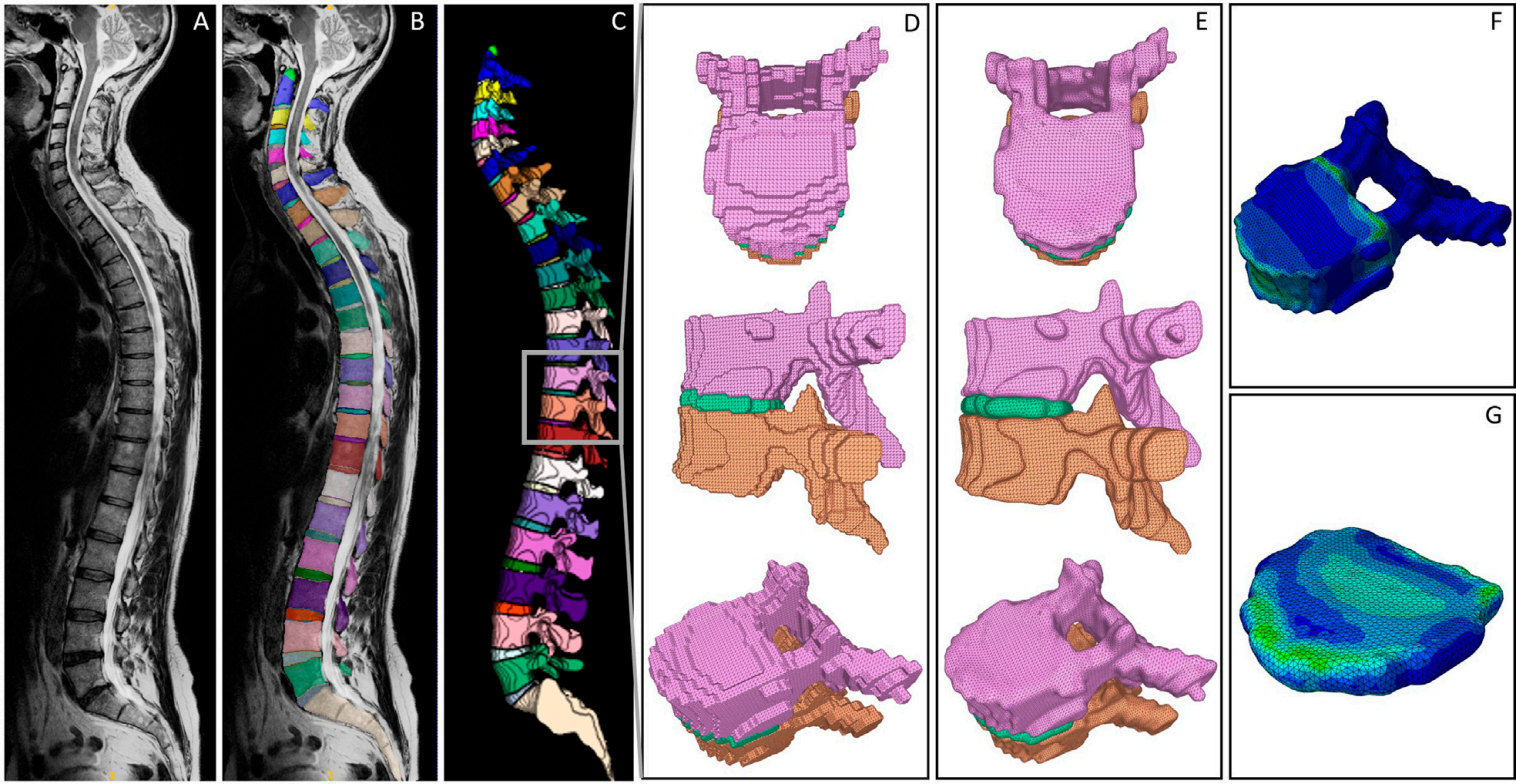
Visualized processing steps in the presented pipeline. **(A)** Exemplary MRI scan. **(B)** Segmentation derived from the SPINEPS network (Moeller et al., 2024). **(C)** 3D representation of the segmentation including a highlighted FSU to demonstrate the initial surface mesh result **(D)**, the smoothed surface meshes **(E)**, and the FEM results of vertebra **(F)** and IVD **(G)**.

We implemented an automated pipeline that takes segmented MRI images of the spine as an input and provides FEM simulation results as an output (Figure 1). All steps were automated using Python as a baseline programming language. We used an in-house dataset that included 30 patients. The data contains MRI images of a 1 × 1 mm resolution in the sagittal plane and a 2.5–3.5 mm slice thickness. The implemented pipeline can broadly be divided into the following substeps: Generation of surface meshes from MRI segmentations, smoothing, mesh filling, volume meshing, FEM modeling and FEM simulation (Figure 2). FE models can be used in a plug-and-play manner to simulate either single bodies, functional spinal units (FSU) or complete spine models. To demonstrate the functionality of the FEM models, we included the automated execution of FEM simulations as the final substep. Therefore, we defined an exemplary load and material model. Note that this work focused on the automation aspect and the resulting FEM stresses and displacements were not interpreted biomechanically. Given that a manual approach would not significantly affect the key pipeline steps—mesh smoothing, volume body creation, or FEM meshing—and that the manual process is highly user-dependent, making precise time comparisons challenging, we chose not to include a traditional manual control group in this study. In what follows, we present a detailed description of the substeps.

### 2.1 MRI to surface mesh

The MRI image segmentation masks were created using SPINEPS, an open-source deep learning approach. Refer to Moeller et al. for details on the segmentation approach (Moeller et al., 2024). In brief, the network segments 14 spinal structures, including vertebrae and IVDs with a dice score above 0.9, respectively. The resulting segmentation masks are visualized in Figures 2B, C. The masks were subsequently edited by removing partial volume segmentations, which were classified by a threshold number of four linked voxels. Segmented partial volumes often lead to sharp edges in the derived surface meshes during smoothing. In vertebrae and IVDs, they were identified by their voxel volume and subsequently removed.

To convert the segmented geometries into a surface mesh, we applied the marching cubes algorithm (van der Walt et al., 2014) with an ascending gradient direction and a step size of 1. Note that for the scope of this work, we considered the segmented endplates to be part of the IVDs by combining their labels during the application of the marching cubes algorithm. As a result, we gained a triangulated mesh of each vertebra and IVD. The resulting meshes of two vertebrae and one IVD (FSU T10-T11) are exemplary shown from the transverse and sagittal view, as well as in an isoparametric angle in Figure 2D.

### 2.2 Smoothing

To eliminate inaccuracies such as stair steps, we applied anatomically constrained smoothing algorithms to the surface meshes of vertebrae and IVDs. Vertebrae were smoothed with a focus on preserving anatomical edges and geometrical characteristics like osteophytes. Shared-node contact surfaces of adjacent vertebrae and IVDs were realized by adaptively smoothing IVDs in a subsequent step. However, both approaches can be divided into three parts: preprocessing, main smoothing and postprocessing.

For vertebrae, preprocessing consisted of mesh repairing steps such as closing holes and concatenating nodes, which were executed using the trimesh Python package. Those steps were required due to inaccuracies in the marching cubes algorithm.

For the selective smoothing step, the mesh vertices of the vertebra were compared to the mesh vertices of the two adjacent IVDs to determine those located on the contact surface, which we here refer to as interface vertices. Contact was assumed for distances below a certain threshold, which was iteratively defined by optical observation. Thresholds ranged between 0.6 and 0.8, depending on the spinal level.

Subsequently, selected interface vertices of the vertebra mesh were smoothed separately using the Laplacian smoothing algorithm (Sorkine, 2005). The smoothed selected vertices are returned to the vertebra mesh before the Taubin smoothing algorithm (Taubin, 1995) was applied to the whole vertebra mesh in a postprocessing step using PyMeshLab (Cignoni et al., 2008). Refer to Nispel et al. for detailed information on the development process and the performance of the smoothing protocol (Nispel et al., 2024).

For the IVDs, preprocessing included Taubin smoothing, Laplace smoothing and mesh repairing steps, which were equal to the ones applied to vertebrae meshes mentioned above. A final dilation step was included to compensate for the volume loss that typically occurs when using Laplace smoothing filters. The subsequent, adaptive smoothing process was aimed at positioning the vertices of the IVD contact surface equivalently to the vertebrae vertices of the contact surface. Simultaneous to the selective smoothing, interface vertices of the IVD were determined using the smoothed, adjacent vertebrae meshes and the above-mentioned distance thresholds. Each interface vertex in the IVD mesh was then replaced with the respectively nearest vertex of the adjacent vertebra mesh. Arisen edges at the borders of the contact area were smoothed in a postprocessing step, which consisted of a Taubin filter and mesh repairing functions. Figure 2E visualizes the final smoothing results for the exemplary FSU.

**FIGURE 3:**
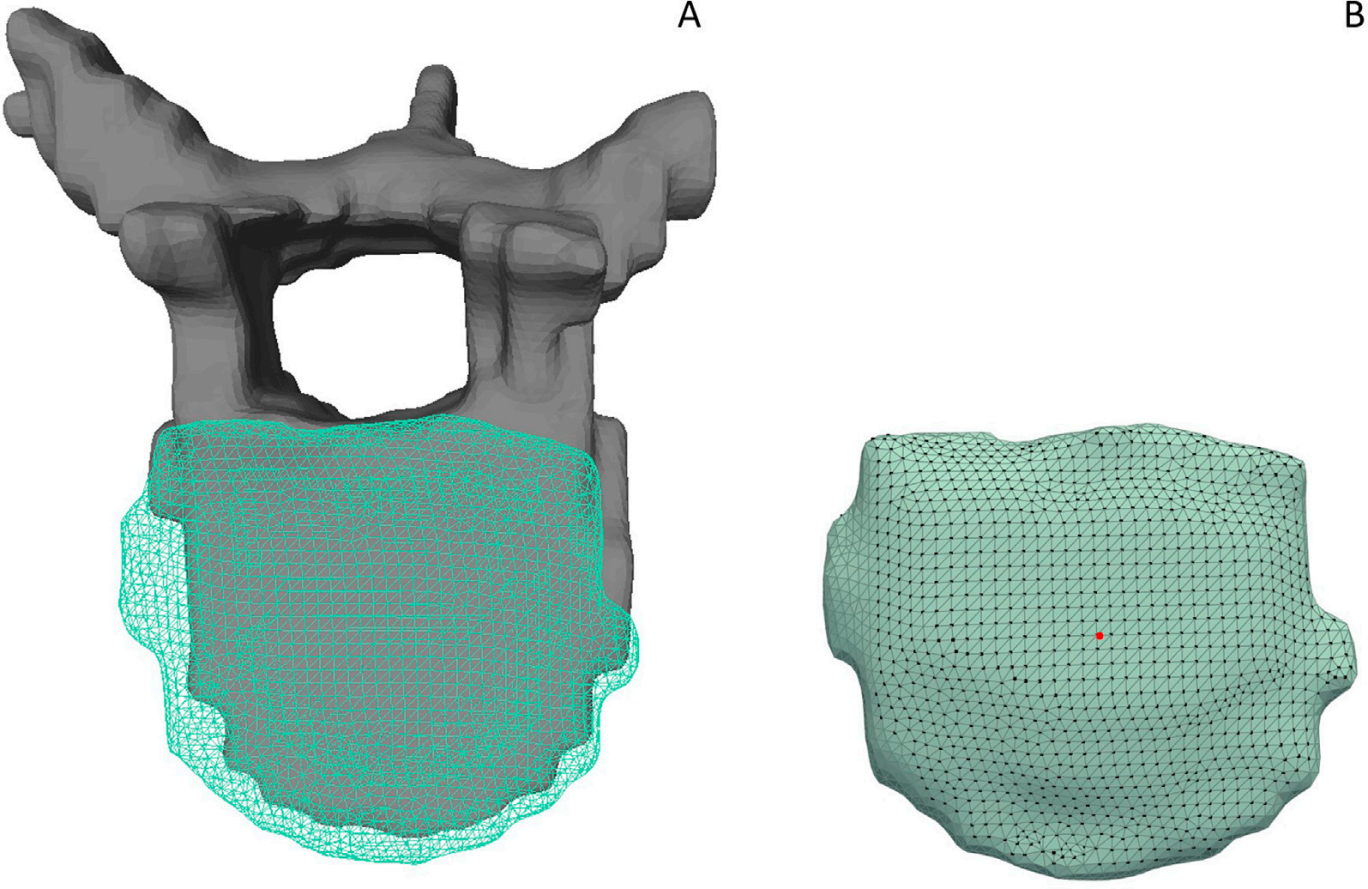
**(A)** Gray, solid vertebra and adjacent IVD displayed as a turquoise grid mesh. **(B)** The respective, selected interface nodes of the IVD model are highlighted. A section without interface points at the left side of the IVD indicates that the adjacent vertebra is not in contact with the disc in this area. The remaining stair step artifacts are still included in the non-contact areas of the model due to the limited spatial resolution of the MR image dataset in the left-right direction.

Note that through this approach, the selected interface vertices represented the anatomical contact surfaces in both IVDs and vertebrae (Figure 3).

### 2.3 Mesh filling

To convert the surface meshes into solid volumes, we applied a surface reconstruction algorithm that transformed the vertices and faces of the triangulated mesh to a continuous surface using the FreeCAD Python interface (Riegel et al., 2024). During this step, a CAD file was created from the meshes. Subsequently, reconstructed surfaces were converted into solid models. In this solid representation, the coordinates of the surface mesh vertices were retained. This step was carried out equivalently for vertebrae and IVDs.

### 2.4 Volume meshing

The resulting solid CAD geometries were subsequently processed using the ABAQUS Python interface. Two equal subprocesses, one for the vertebrae and one for the IVDs, were carried out. The subprocesses primarily involved the consecutive, homogeneous meshing of the generated CAD geometries. We used tetrahedral elements (C3D10), as they are able to represent the vertex positions of the initial surface mesh. Geometries were seeded with a global seed size of 1 mm, along with a deviation factor and a minimum element size, both set to 0.1 mm. The created, meshed part was stored in an ABAQUS input file, including the name of the respective vertebra or IVD.

### 2.5 Volume mesh to FEM model

To accurately represent the anatomical loading situation within a simulation framework, load should be distributed only among the nodes that are in contact with the adjacent body. To realize this, we included the definition of node sets in the generation of the FEM models. Each part, vertebra or IVD, therefore contained two node sets, one for the superior surface and one for the inferior surface, respectively. To define the node sets for the vertebrae, we first parsed the volume nodes that were generated in the volume meshing step. Note that the indices of the vertices changed during the conversion of the surface mesh to the volume mesh. Iterating through the interface nodes defined in the smoothing step allowed us to find the respective nodes in the volume mesh by a comparison of their rounded coordinates. The corresponding volume node indices were appended to the respective node set.

For both, vertebrae and IVDs, we additionally included two node sets containing one superior and inferior reference node, respectively. The reference node was defined by averaging all surface nodes and determining the closest node to the resulting average.

To finalize the simulation definition of the FEM parts, we implemented an automatic inclusion of the remaining simulation parameters, namely material parameters, boundary conditions, loading and constraints.

**FIGURE 4:**
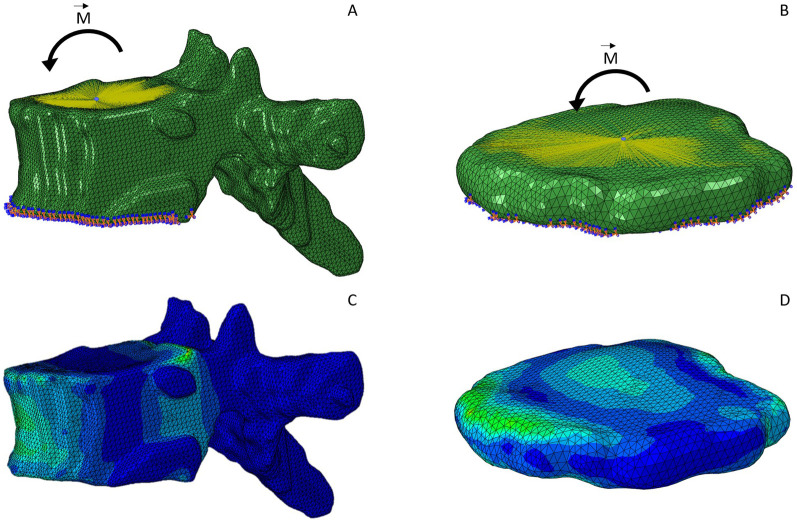
FEM simulation model of an exemplary vertebra **(A)** and IVD **(B)**. The kinematic coupling constraint on the superior endplate surface is indicated with the yellow lines pointing towards a reference node at the center of the surface. A flexion moment was applied at the center of the surface. The inferior surface is constrained in all six DoF, indicated by the orange and blue symbols. **(C, D)** display the same two models after simulation, which is complemented by the color grading indicating the von Mises stress.

For each standalone FEM model of either a vertebra or an IVD, a kinematic coupling constraint was implemented to create a rigid body at the superior surface. The coupled surface was defined using the superior surface node set, which represented the biological contact area of the vertebra or IVD and its respective adjacent IVD or vertebra, respectively. The reference node was taken from the previously defined reference node set. For all vertebrae and IVDs, we simulated a flexion moment of 7.5 Nm (Dreischarf et al., 2014). The moment was applied to the reference node of the superior endplate surface. As a boundary condition, the inferior node set was restrained in all six degrees of freedom (DoF). Figures 4A, B show the FEM simulation models including boundary conditions, constraints and load cases for an exemplary vertebra and IVD.

Since this work focused on the automation methodology in the first place, simplified material definitions were employed for the FEM simulation. Unlike the complex IVD physiology, which includes a gel-like core and multiple layers of concentric fibers, all parts were meshed uniformly in this work, including a uniform material definition. Linear elastic, isotropic material parameters were calculated based on the literature, both for vertebrae (Bouzakis et al., 2004; Zhou et al., 2000; Bruno et al., 2014) and IVDs (El Bojairami et al., 2020).

To finalize the FEM simulations, we defined a static analysis and appended the simulation files as separate entities to a batch file for automatic execution. Simulation results (Figures 4C, D) were visually inspected.

### 2.6 Evaluation of the pipeline

The whole pipeline was executed on an i7 CPU machine with 8 cores, 64 GB RAM and a Windows operating system. We utilized Python versions 3.8 and 3.10, the latter in an Anaconda environment, as well as ABAQUS for the FEM simulation. The pipeline execution was limited to running on a single core to standardize the computational process.

To quantify the performance of our pipeline, we analyzed the mesh quality of the generated FEM meshes. This was done by parsing the tetrahedral elements of the meshes and querying their aspect ratio. We considered the aspect ratio to be the relation of the elements’ maximum and minimum edge lengths. Elements with an aspect ratio >5 were considered of poor quality (Campbell and Petrella, 2015; Lavecchia et al., 2018). Note that, as a guideline, FEM meshes are typically aimed at less than 10% poor-quality elements to minimize the potential impact on simulation accuracy and convergence (Neves et al., 2018). For each generated FEM part, the percentage of poor quality elements was calculated. For the processed dataset of 30 patients, we averaged the percentage of poor quality elements for each vertebra and IVD, respectively.

For further performance evaluation, we reviewed the number of FEM models for which solver convergence was achieved during simulation, contrasting successful attempts with those that failed. In addition, we visually evaluated the reasons for the failed attempts.

Finally, we compared the calculation time needed for each part of the pipeline: MRI to surface mesh, smoothing, filling, volume meshing, volume mesh to FEM Model, and lastly, the FEM simulation. We thereby distinguished between the duration needed for the vertebrae and the IVDs. The resulting durations were statistically evaluated across the 30 subjects that were analyzed. We addressed failures in the pipeline by identifying and registering the respective vertebra or IVD and subsequently conducting visual analysis to determine the cause of failure.

## 3 Results

We implemented a pipeline that is able to automatically calculate FEM results of vertebrae and IVDs in large cohorts. The pipeline is based on an automated segmentation of vertebrae and IVDs in MRI images (Moeller et al., 2024). From this, surface meshes were derived and selectively smoothed to mimic the biological endplate shapes, compensating for image resolution inaccuracies. Hereby, we take advantage of the MRI segmentations by including both bone and soft tissue. Smoothed surface meshes were then automatically transformed to FEM volume meshes, which contain individual node sets of the superior and inferior contact surface to the adjacent vertebra or IVD, respectively. FEM models were available as a combination of nodes and elements. In our study, these models were supplemented with boundary conditions, loading and material definition to create and simulate FEM models. Using this pipeline, we processed all 30 patient MRI scans, resulting in a total of 637 vertebrae FEM simulations and 600 IVD FEM simulations.

### 3.1 Mesh quality

Vertebrae and IVD meshes differed significantly in their numbers of nodes and elements (Table 1). On average, vertebrae meshes contained approximately 229.500 elements, 4.5 times more than the average IVD mesh.

**TABLE 1:** Number of nodes and elements for all IVD and vertebra FEM meshes generated by the pipeline.

	Nodes			Elements		
	mean	max	min	mean	max	min
IVDs (n = 593)	9.811	36.494	939	50.414	196.357	3.756
Vertebrae (n = 637)	43.848	98.884	1.646	228.757	529.102	7.435

**FIGURE 5:**
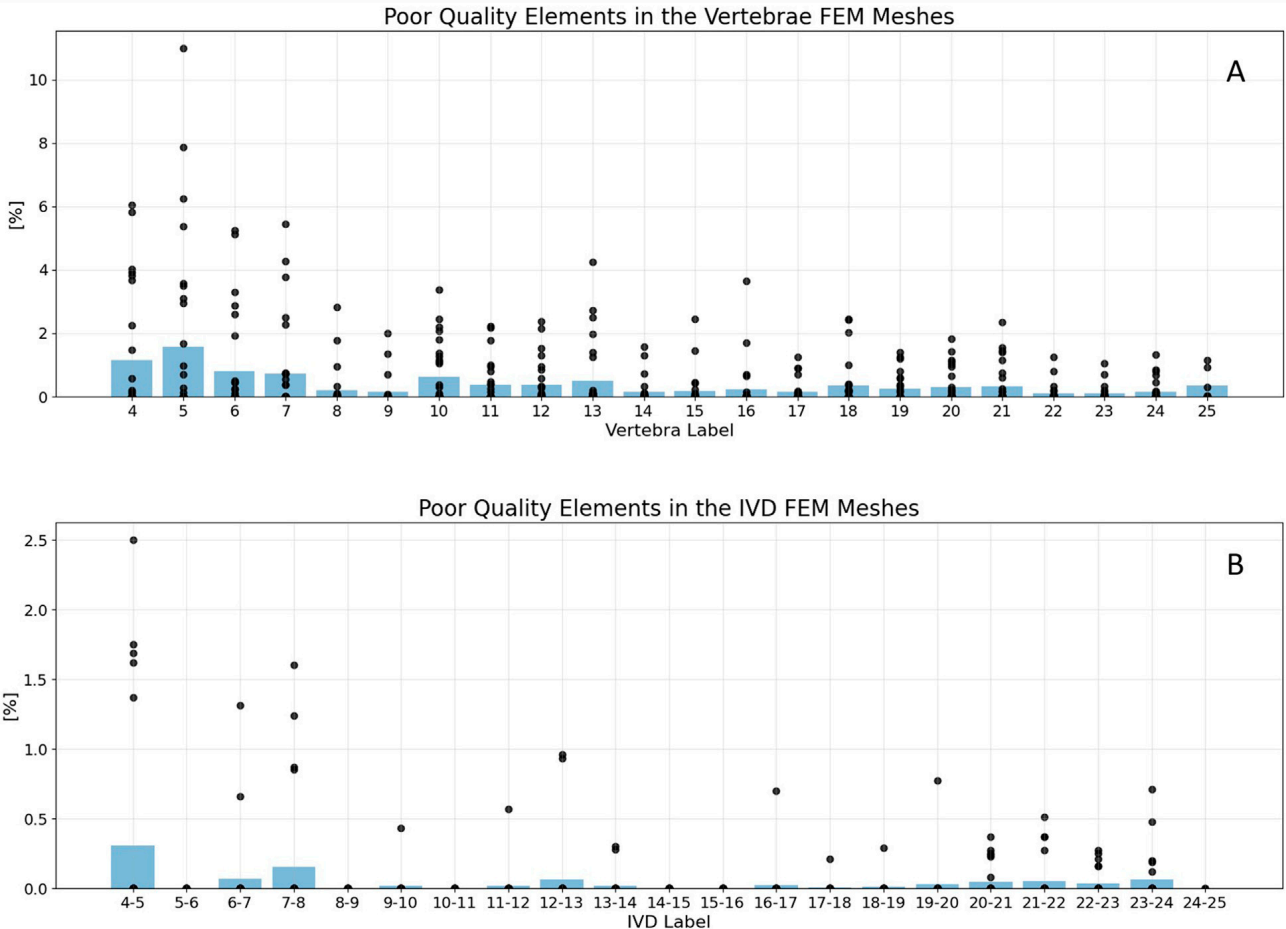
Mesh statistics for the generated vertebrae **(A)** and IVD FEM meshes **(B)**. Black points represent the percentage of poor quality elements in single FEM models, with poor quality being defined as an aspect ratio >5. The blue beams indicate the average percentage of poor quality elements in all models of the specific label. Failed vertebrae models were excluded from the plot. For vertebrae meshes **(A)**, the maximum share of poor quality elements was 13% in a C5 vertebrae. The average percentage of poor quality elements was never below 2% in both, vertebrae and IVDs. For IVDs **(B)**, the average percentage of poor quality elements was never even below 0.5%. The lowest mesh quality was generated in the cervical spine.

Across all created FEM models, both vertebrae and IVDs, the average percentage of poor quality elements remained below 2%, well within the 10% guideline defining a good quality mesh (Figure 5). Evaluations were conducted label-wise across the entire dataset, with each label corresponding to a specific spine level. Consequently, vertebra labels ranged from 4 to 25, while IVD labels ranged from 4-5 to 24-25.

Specifically for vertebrae meshes (Figure 5A), 98.6% of models contained even less than 5% elements of poor quality. The highest proportion of poor quality elements, reaching 13%, was observed in a label 5 vertebra (C5). It is represented as the highest outlier in Figure 5A.

Regarding IVDs (Figure 5B), the average percentage of poor quality elements never dropped below 0.5%. This indicates a good mesh quality, well within the defined guideline for mesh quality (10%). In the whole dataset, six IVD labels even contained no elements with poor quality, which were 5-6, 8-9, 10-11, 14-15, 15-16, as well as 24-25. For another six labels, namely 9-10, 11-12, 16-17, 17-18, 18-19 and 19-20, only one IVD model was generated which contained elements of poor quality.

For both vertebra and IVD models, the lowest mesh quality was consistently found in the cervical spine. The best results considering mesh quality were achieved in the higher labels, namely the lumbar spine.

### 3.2 Failed attempts

Among all 637 vertebra and 600 IVD simulations, ten vertebrae and seven IVDs raised an error within the process, which led to the incompletion of the FEM simulation. Errors were either caused by the poor quality of the segmentation mask of the respective body, or arose during the smoothing process. Smoothing errors were caused by a low surface mesh quality, including either non-manifold edges or holes, which was mainly the case for IVDs. Segmentation errors appeared when disconnected volumes were present, which was only the case for vertebrae.

### 3.3 Calculation times

With an average number of 21 vertebrae and 20 IVDs per subject, the average processing time was 4.4 min for a vertebra and 31 s for an IVD. For one subject, the average duration to process and simulate all vertebrae was 93.3 min. For all IVDs, the average calculation time per subject was 10.4 min.

**FIGURE 6:**
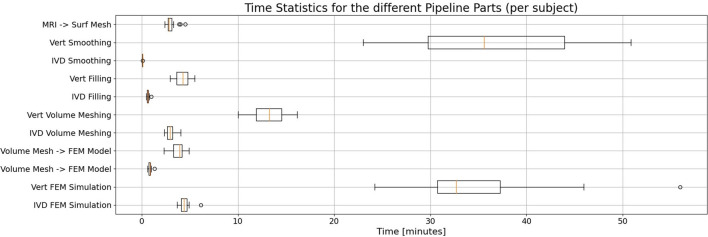
Statistical evaluation of the duration needed for the different parts of the pipeline when processing a single subject, displayed in a box plot. Whisker boundaries are drawn at 1.5 times the interquartile range (IQR). Outliers are marked by single points. Each part’s duration is given for the vertebrae and the IVDs, respectively. Note that with considerable margin, the vertebrae smoothing and FEM simulation take the most time in the automated pipeline.

With significant distance, vertebrae smoothing and their FEM simulation make up the most costly parts of the pipeline considering processing times (Figure 6). Note that especially for the smoothing process, the selective smoothing of vertebrae takes an average time of 35 min per subject, while the adaptive IVD smoothing algorithm only takes less than a minute (Table 2). With approximately 13 min per subject, meshing the vertebrae volumes is still among the most time-consuming parts of the pipeline. Note that the MRI to surface mesh process includes the complete processing and segmentation of the MRI file, as well as alignment and the determination of points of interest.

**TABLE 2:** Quantitative statistical values for the durations of different pipeline parts. The averages refer to the duration needed to process one subject.

Pipeline part	Average duration [s]	Min duration [s]	Max duration [s]
MRI → Surf Mesh	178.15	144.34	272.09
Vert Smoothing	2,195.24	1,381.07	3,050.03
IVD Smoothing	4.23	3.47	5.53
Vert Surface Mesh → Volume Mesh	253.59	176.69	330.53
IVD Surface Mesh → Volume Mesh	38.32	29.15	58.22
Vert Volume Mesh → inp file part	791.39	602.23	970.29
IVD Volume Mesh → inp file part	178.82	140.76	243.29
Vert inp file part → inp file model	224.97	139.18	293.58
IVD inp file part → inp file model	50.81	37.46	79.82
Vert FEM Simulation	2,046.73	1,452.35	3,358.66
IVD FEM simulation	265.57	219.22	368.09

Finally, the developed pipeline is automated to a point where only the MRI image paths of the patients and the FEM simulation parameters need to be defined as input. The latter includes loading and material parameters. As output, an ABAQUS output database is created, containing deformation and stress values as requested. No manual steps were required.

## 4 Discussion

637 vertebrae and 600 IVDs were modeled and simulated using our automated pipeline, with an average duration of 4.4 min per vertebra and 31 s per IVD. We evaluated the quality and performance of the pipeline by investigating the quality of generated meshes, failed attempts and processing times.

With the predefined mesh quality criteria, more than 98% of generated models achieved good quality. This was also reflected in the low amount of failed attempts - only ten vertebrae and seven IVDs raised an error within the modeling and simulation process. A detailed analysis of these failed attempts revealed insights into the failure mechanisms in our pipeline. For all cervical IVDs, errors were due to self-intersecting faces or non-manifold edges in the surface mesh, which occurred in regions of small segmentation volumes. Failure mechanisms in thoracic IVDs were connected either to a hole in the raw mesh or small segmentation volumes. In one case, a hole led to a T-vertex in the presmoothed mesh, which caused the self-intersection of faces in the adaptively smoothed IVD. In the only failed lumbar IVD, T-vertices were created for an unidentified reason regarding the good quality of the raw mesh. We additionally monitored all failed vertebrae for apparent quality issues, and found similar failure mechanisms as in the IVDs. In six of the ten vertebrae, self-intersecting faces were found, which led to failing FEM mesh generation. In the remaining cases, mostly cervical vertebrae, the segmentation mask contained two disconnected volumes. Our pipeline was able to generate two disconnected surface mesh parts from these segmentation masks. However, only one volume was converted to a solid mesh. We consider this an issue of the segmentation mask and not primarily a limitation of the here presented pipeline.

The processing time for the FEM simulation of vertebrae was approximately tenfold higher compared to that for IVDs. It is worth noting that meshing and smoothing times are directly proportional to the number of elements in the mesh, which is roughly 4.5 times greater in vertebrae models than in IVD models.

Assessing the efficiency of our approach versus traditional manual methods is challenging due to the lack of qualitative literature data on the time required for manual implementation. Manual processes typically involve segmentation software for surface mesh derivation, followed by volumetric model generation and FEM simulations using separate tools, a process that can take up to several days (Caprara et al., 2021). In contrast, our automated pipeline drastically reduces this timeframe. With no user interaction and reliance solely on an MRI image, our method achieves biomechanical analysis of a vertebra or IVD within just about 4.3 min or 27 s, respectively. In addition, it is insusceptible to variability due to manual steps.

One limitation of our pipeline is the homogeneous, isotropic modeling approach, that is in conflict with recent IVD and vertebra models, which contain anisotropic material definitions in the case of vertebrae (Molinari and Falcinelli, 2021; Fleps and Morgan, 2022), and biphasic, hyperelastic or fiber-reinforced components in the case of the IVD (Gruber et al., 2024; Dreischarf et al., 2014).

For the IVD, these more complex modeling approaches go hand in hand with anisotropic meshing that comprises, in the best case, hexahedral elements (Dreischarf et al., 2014). The presented approach does not enable a structured mesh including multiple phases, such as e.g. a nucleus pulposus cavity or annulus fibrosus fibers. Besides the lack of detail this brings to the resulting mechanics, calculation times would likely increase when using a more realistic material definition. However, experiments indicate that already in mildly degenerated discs, fluid content decreases significantly (Galbusera et al., 2014; Iatridis et al., 1998; Iatridis et al., 1997; Johannessen and Elliott, 2005). As a result, tissue behaved almost linearly and a change from anisotropic to isotropic nature was reported (Iatridis et al., 1998). For lumbar IVDs, a slowly increasing loss of flexibility correlates with an increasing grade of degeneration (Kettler et al., 2011). Interestingly, studies have found an abrupt decrease in flexibility for mildly degenerated thoracic discs with Pfirrmann grade (Pfirrmann et al., 2001) of 1 (Liebsch and Wilke, 2022) as opposed to healthy discs. Precisely, the tetrahedral representation of IVDs, as it results from our pipeline, might be sufficient for analyzing further degenerated IVDs with the advantage of low complexity and comparably short computation time. In addition, biomechanical FEM studies of multiple FSUs that investigate the holistic behavior of the spine, like the one conducted by El Bojairami et al. (2020), may profit from the reduced complexity of the IVD mesh. To particularly investigate the advantages of a more complex IVD model in their use case, El Bojairami et al. implemented an additional two-phase fluid-structure model besides their isotropic one. And extracted the hydrostatic pressure from an enclosed, hydrostatic pressure element within the nucleus pulposus cavity (El Bojairami et al., 2020). The comparison between the two modeling approaches (isotropic vs. complex) showed that both methods produced results that overlapped to a great extent, with a maximum discrepancy of approximately 4% at certain flexion angles. Moreover, tetrahedral elements allow for a more straightforward implementation of shared-node contact surfaces, which is beneficial for computation times and numerical stability (El Bojairami et al., 2020). A recent observation in FEM simulations of degenerated discs even found that tetrahedral elements were more stable and accurate in the results (Fasser et al., 2022). Altogether, these findings may support employing tetrahedral elements and isotropic material properties for the IVD in limited circumstances, particularly when considering factors such as computational efficiency and simplicity of implementation. However, multiple studies have analyzed the benefits of biphasic or fiber-reinforced material models for the IVD (Gruber et al., 2024; Stadelmann et al., 2018; Knapik et al., 2022; Sciortino et al., 2023). A differentiated consideration of modeling approaches is crucial, and their application should be carefully selected depending on the specific research question.

Apart from the IVD model complexity, vertebrae models in our pipeline are also limited to isotropic, tetrahedral meshes, which lack the distinction between cortical and cancellous bone. However, the inclusion of shell elements could easily be realized similarly to the approach of Imai et al., who attached a 0.4 mm thick layer of shell elements to the outer surface of the tetrahedra (Imai, 2015). Nevertheless, the application of modeling approaches should also be carefully selected based on the specific research question. By providing the pipeline to the public and ensuring the output of a standardized mesh representation as an .inp file, research groups are enabled to further process and adapt meshes of their own datasets towards their requirement.

In comparison to morphing approaches for FEM model generation (Campbell and Petrella, 2015; Lavecchia et al., 2018; Caprara et al., 2021; Fasser et al., 2022; Castro-Mateos et al., 2015; Caprara et al., 2021; Fasser et al., 2022), the here presented filling approach represents more details of the segmentation, which unfortunately includes low-resolution features like stair steps as well. To counter this, we implemented a tailored smoothing algorithm Nispel et al. (2024). However, since the focus was placed on smoothing the interface surfaces, the circumferential surfaces of vertebrae and IVDs were only considered during the pre- and postprocessing of smoothing, resulting in stair-step artifacts in the final meshes. This may be viewed as a limiting factor of our approach. With advancements in faster MRI scanning methods, higher-resolution scans will become more readily available, potentially reducing these stair-step artifacts in the circumferential surfaces. Considering the final FEM mesh quality, we do not expect any improvement in the FEM mesh with the enhancement of MRI resolution, as the size of the FEM elements is much smaller than the stair-step artifacts.

Finally, given the MRI segmentation in our pipeline, hard tissue prediction is not as stable as in CT image segmentation. Potential errors in correctly predicting detailed vertebrae characteristics like osteophytes could be propagated to the FEM results and may affect the result quality negatively.

## 5 Conclusion and outlook

The goal of this work was to advance the automated generation of patient-specific FEM models derived directly from MRI segmentations. In comparison to recent approaches, our approach is based on the MRI image itself and does not rely on templates like SSMs. The process of morphing a mesh is thereby obsolete, which constitutes the advantage of having fewer processing steps and higher flexibility in representing geometries that do not fit into statistical norms, like osteophytes, strongly deformed vertebrae or extreme bulges in IVDs.

By realizing surfaces with shared nodes and elements for adjacent vertebrae and IVDs, we spare the time-consuming and unstable process of implementing penalties during contact modeling. In addition, shared interface nodes are advantageous in the framework of exchanging load and displacement data in coupled MBS and FEM simulations.

Manual implementations of full spine models may be subject to automation efforts in the future. Constitutive models of the IVD and vertebrae can be advanced by including locally varying, individualized MRI- or CT-derived material parameters. To include both detailed bone geometries with, e.g., osteophytes and IVD deformities such as bulges, a co-registration of MRI and available CT data could be beneficial.

For the IVD, a differentiation between nucleus pulposus and annulus fibrosus, together with a fiber-reinforced or biphasic implementation could be beneficial in answering specific research questions. Vertebrae might benefit from a differentiation between cortical shell and trabecular bone. Prospectively, combining our pipeline with validated models, as demonstrated in our group’s recent work Gruber et al. (2024), will enable large cohort studies to gain insight into the causes of spinal disorders such as degeneration or back pain.

## Data Availability

The raw data supporting the conclusion of this article will be made available by the authors, without undue reservation.
